# Strong and widespread cycloheximide resistance in *Stichococcus*-like eukaryotic algal taxa

**DOI:** 10.1038/s41598-022-05116-y

**Published:** 2022-01-20

**Authors:** Nur Hidayu Syuhada, Faradina Merican, Syazana Zaki, Paul A. Broady, Peter Convey, Narongrit Muangmai

**Affiliations:** 1 School of Biological Sciences, Universiti Sains Malaysia, Gelugor, Penang Malaysia; 2 National Antarctic Research Centre, University of Malaya, Kuala Lumpur, Malaysia; 3 School of Biological Sciences, University of Canterbury, Christchurch, New Zealand; 4 British Antarctic Survey, Cambridge, UK; 5 Department of Zoology, University of Johannesburg, Johannesburg, South Africa; 6 Department of Fishery Biology, Faculty of Fisheries, Kasetsart University, Bangkok, Thailand; 7 Graduate School of Integrated Sciences for Life, Hiroshima University, Hiroshima, Japan

**Keywords:** Microbiology, Environmental sciences

## Abstract

This study was initiated following the serendipitous discovery of a unialgal culture of a *Stichococcus*-like green alga (Chlorophyta) newly isolated from soil collected on Signy Island (maritime Antarctica) in growth medium supplemented with 100 µg/mL cycloheximide (CHX, a widely used antibiotic active against most eukaryotes). In order to test the generality of CHX resistance in taxa originally identified as members of *Stichococcus* (the detailed taxonomic relationships within this group of algae have been updated since our study took place), six strains were studied: two strains isolated from recent substrate collections from Signy Island (maritime Antarctica) (“Antarctica” 1 and “Antarctica” 2), one isolated from this island about 50 years ago (“Antarctica” 3) and single Arctic (“Arctic”), temperate (“Temperate”) and tropical (“Tropical”) strains. The sensitivity of each strain towards CHX was compared by determining the minimum inhibitory concentration (MIC), and growth rate and lag time when exposed to different CHX concentrations. All strains except “Temperate” were highly resistant to CHX (MIC > 1000 µg/mL), while “Temperate” was resistant to 62.5 µg/mL (a concentration still considerably greater than any previously reported for algae). All highly resistant strains showed no significant differences in growth rate between control and treatment (1000 µg/mL CHX) conditions. Morphological examination suggested that four strains were consistent with the description of the species *Stichococcus bacillaris* while the remaining two conformed to *S. mirabilis*. However, based on sequence analyses and the recently available phylogeny, only one strain, “Temperate”, was confirmed to be *S. bacillaris*, while “Tropical” represents the newly erected genus *Tetratostichococcus*, “Antarctica 1” *Tritostichococcus*, and “Antarctica 2”, “Antarctica 3” and “Arctic” *Deuterostichococcus*. Both phylogenetic and CHX sensitivity analyses suggest that CHX resistance is potentially widespread within this group of algae.

## Introduction

Resistance to antibiotic agents is an ancient and widespread phenomenon in the natural environment^1^. Its evolution is stimulated by the selection pressure of sharing an ecological niche with an antibiotic-producing organism^2^. An organism is considered naturally resistant to an antibiotic agent when it has developed a mechanism to mitigate the toxic effects of that agent and continues to function in its presence^3^. Organisms that were originally susceptible to an agent may also later acquire resistance through mechanisms such as chromosomal mutation or by acquisition from external genetic elements obtained from naturally resistant organisms present in the environment^3^.

Cycloheximide (CHX) is an antibiotic originally discovered in studies of the bacterium *Streptomyces griseus*^4^, where it was found to be effective in killing fungal pathogens at a concentration as low as 0.2 µg/mL but possessed little or no antibiotic activity against bacteria^4^. CHX inhibits the growth of most eukaryotes by interfering with 80S ribosomes during protein synthesis^5^. Studies using cultures have confirmed the antibiotic activity of CHX against a range of eukaryotic algae and fungi and that it has little or no effect on prokaryotes^6–9^. CHX completely inhibited the growth of four of 10 species of Chlorophyta at concentrations as low as 1 µg/mL and none were tolerant to more than 50 µg/mL^8^. At concentrations of 20 µg/mL or less, CHX inhibited the growth of yellow-green algae and diatoms (Ochrophyta (= Heterokontophyta))^8^. Complete lysis of cells of *Euglena gracilis* occurred within seven days in broth containing 100 µg/mL CHX^9^. These investigations have led to the standard and widespread use of CHX at 20–200 µg/mL in bacterial and cyanobacterial cultures to eliminate eukaryotic algae and fungi^10–14^ against which it is regarded as one of the most effective antibiotics^9^.

Resistance to CHX has now been reported in some groups of yeasts, where it has also been used as taxonomic marker^15^. Cloning of the CHX resistance gene from a naturally resistant strain of yeast into a sensitive strain has provided a convenient dominant vector marker for recombinant DNA technology^16,17^. Naturally occurring resistance to CHX in yeasts is known in *Saccharomyces* (200 µg/mL) and *Kluyveromyces* (500 µg/mL)^18^. In contrast, CHX resistance has been recorded in only one wild-type alga, the unicellular rhodophyte *Cyanidioschyzon merolae*, which was isolated from an acidic hot spring^19^. This was resistant to only very low CHX concentration (0.5 µg/mL) after an extended lag time in culture of up to 10 days.

Polar microalgae in terrestrial habitats can be exposed to harsh environmental conditions such as freezing temperatures, low water availability, and continuous daylight during summer and darkness during winter^20^. Antarctic green algae have evolved both avoidance and protection/resistance strategies, as well as mechanisms for repair of damage, which enable them to tolerate these extremes^21,22^. However, to our knowledge, research has yet to address the adaptive strategies developed by polar microalgae in the presence of natural antibiotic compounds, despite the presence in Antarctic soils of compounds that can inhibit growth of eukaryotes^23^.

In an initial isolation of cyanobacteria from samples obtained from Signy Island, South Orkney Islands, Antarctica, we observed growth of a small number of discrete colonies of green algae on multiple culture plates of agarised BBM supplemented with 100 µg/mL CHX. Based on light microscopy examination of morphological features, these were identified as a *Stichococcus*-like alga. This study set out to confirm the identity of these algae and the presence of resistance to CHX, and to extend the number of strains studied in order to assess how widespread resistance is within other representatives assigned at the time to this genus. The fitness of algae in the presence of CHX was measured by assessing the Minimum Inhibitory Concentration (MIC), one of the most commonly used methods of quantifying microbial fitness^24^. Growth rate and lag period of cultures in different CHX concentrations were also measured and compared. As morphological variability in *Stichococcus* is very low^25–27^, morphological assessment was combined with molecular phylogenetic analyses to confirm the generic identity of the strains examined here. We note that, subsequent to this study being carried out, a new molecular phylogenetic analysis has become available that has erected several new genera within the original genus ‘*Stichococcus*’^28^.

## Results

### The minimum inhibitory concentration (MIC) of cycloheximide

Visible green growth was observed at up to the maximum tested CHX concentration (1000 µg/mL) for all strains except “Temperate” (Table 1). The control *Chlorella* and *Coccomyxa* strains were the least resistant. Visual observations were supported by growth assessment as indicated by both chlorophyll fluorescence and expressed as cell density using regression analysis. The scatterplots (Fig. S1) showed a strong positive linear relationship between chlorophyll fluorescence and cell density in all tested strains, which was confirmed by Pearson’s correlation analysis (Table S1).

**Table 1:** Minimum inhibitory concentrations (MIC) of cycloheximide for each of six strains of *Stichococcus*-like algae and two control green algal strains assessed by visual observation on Day 14 of growth in culture.

Strains	CHX concentration (µg/mL)
***Stichococcus*-like**	
“Antarctica 1”	> 1000
“Antarctica 2”	> 1000
“Antarctica 3”	> 1000
“Arctic”	> 1000
“Tropical”	> 1000
“Temperate”	62.5
**Controls**	
*Chlorella*	31.3
*Coccomyxa*	15.6

**Figure 1:**
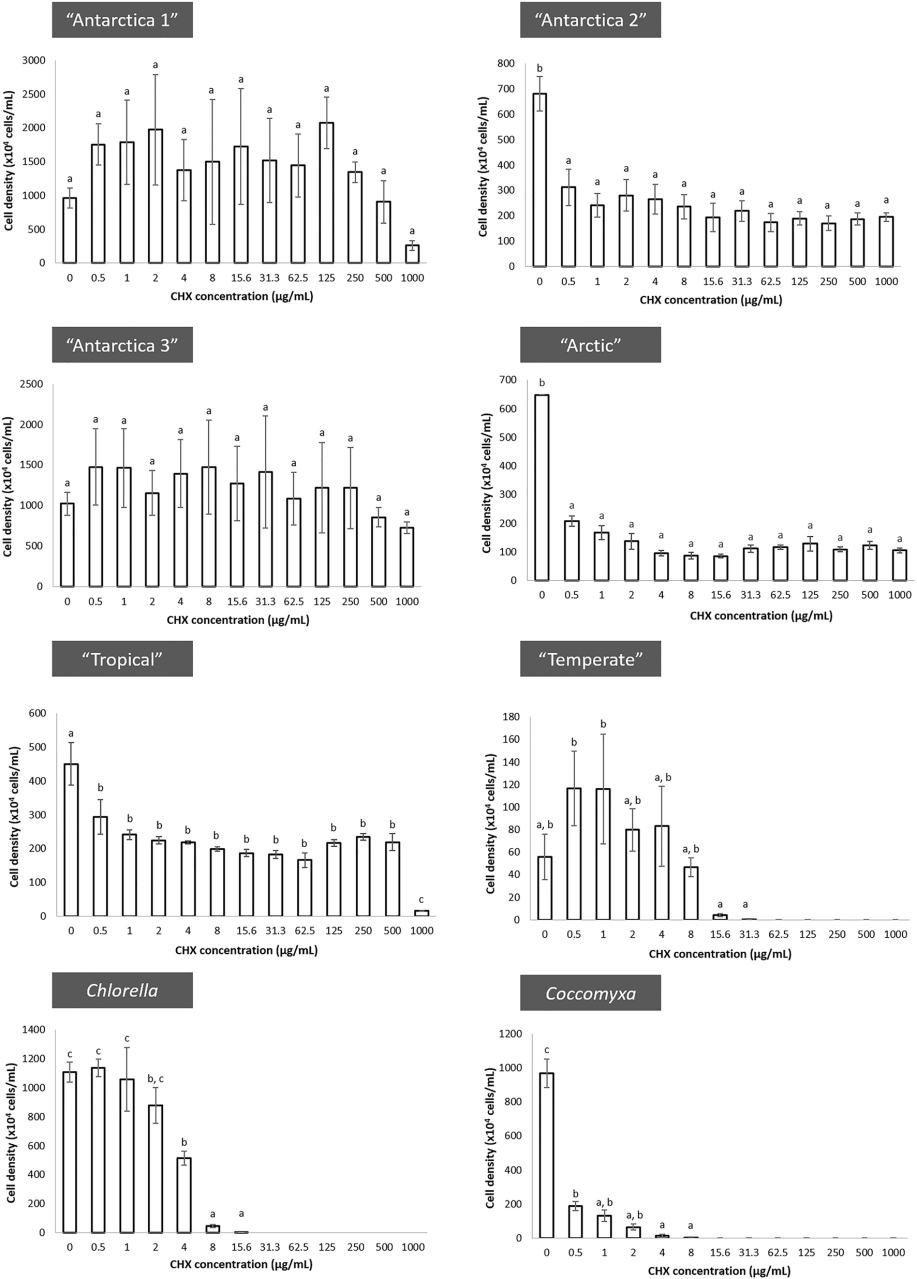
Mean (± standard error) of cell density achieved by each of six strains of *Stichococcus*-like algae and single strains of *Chlorella* and *Coccomyxa* in BBM liquid medium supplemented with different CHX concentrations after two weeks’ incubation. Means sharing the same letter (a, b or c) are not significantly different (Tukey’s HSD, *p* < 0.05).

The MIC values of the susceptible strains (“Temperate”, *Chlorella* and *Coccomyxa*) were determined by the null value of chlorophyll fluorescence. Figure 1 shows the mean cell density achieved by each studied strain exposed to different CHX concentrations after incubation for 2 weeks. One-way ANOVA with post hoc Tukey’s pairwise comparisons indicated no significant differences in the cell densities achieved between all CHX concentrations including the control for strains “Antarctica 3” and “Antarctica 1”, indicating that exposure to CHX had no negative effect on growth for these strains. The cell densities were significantly different between the controls and the treatments for strains “Antarctica 2” and “Arctic” but no differences were detected between the different concentrations of CHX. A similar trend was also seen in strain “Tropical”, except that 1000 µg/mL CHX had a significantly greater negative effect on growth. No growth was recorded at or above 62.5 µg/mL CHX, 31.3 µg/mL CHX and 15.6 µg/mL CHX in “Temperate”, *Chlorella* and *Coccomyxa*, respectively, consistent with the visual MIC result.

### Growth rate assessment

**Figure 2:**
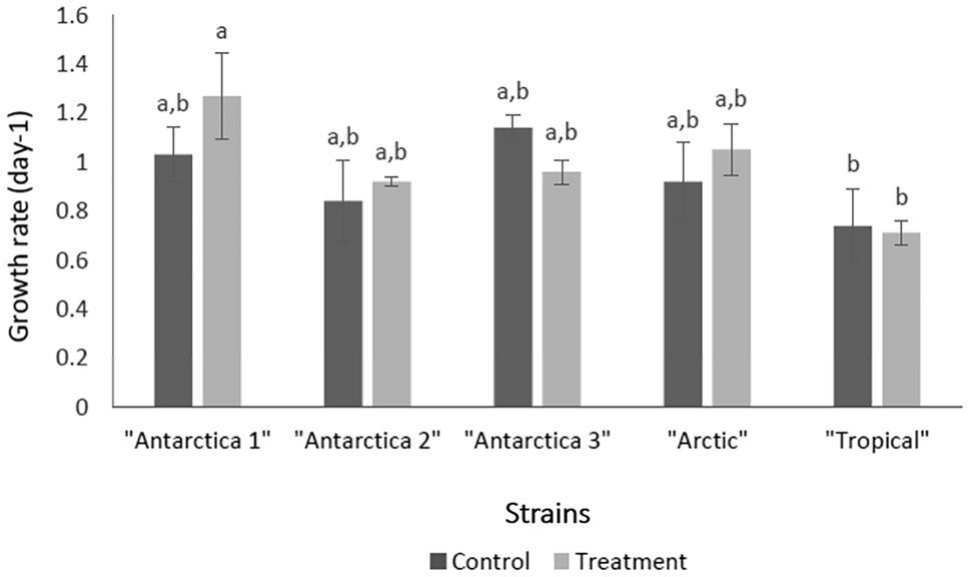
Mean (± standard error) of the growth rate of each of five strains of *Stichococcus*-like algae in control (BBM) versus BBM + 1000 µg/mL CHX. Means sharing the same letter (a, b) are not significantly different (Tukey’s HSD).

The growth rates achieved by each strain in BBM (control) and BBM + 1000 µg/mL (treatment) CHX are presented in Fig. 2. One-way Welch’s ANOVA indicated there was a significant difference in the growth rates achieved between the strains. Based on Games-Howell post-hoc test, there was a significantly greater growth rate in “Antarctica 1” compared with the “Tropical” strain control and treatment growth rates (Table S2).

**Figure 3:**
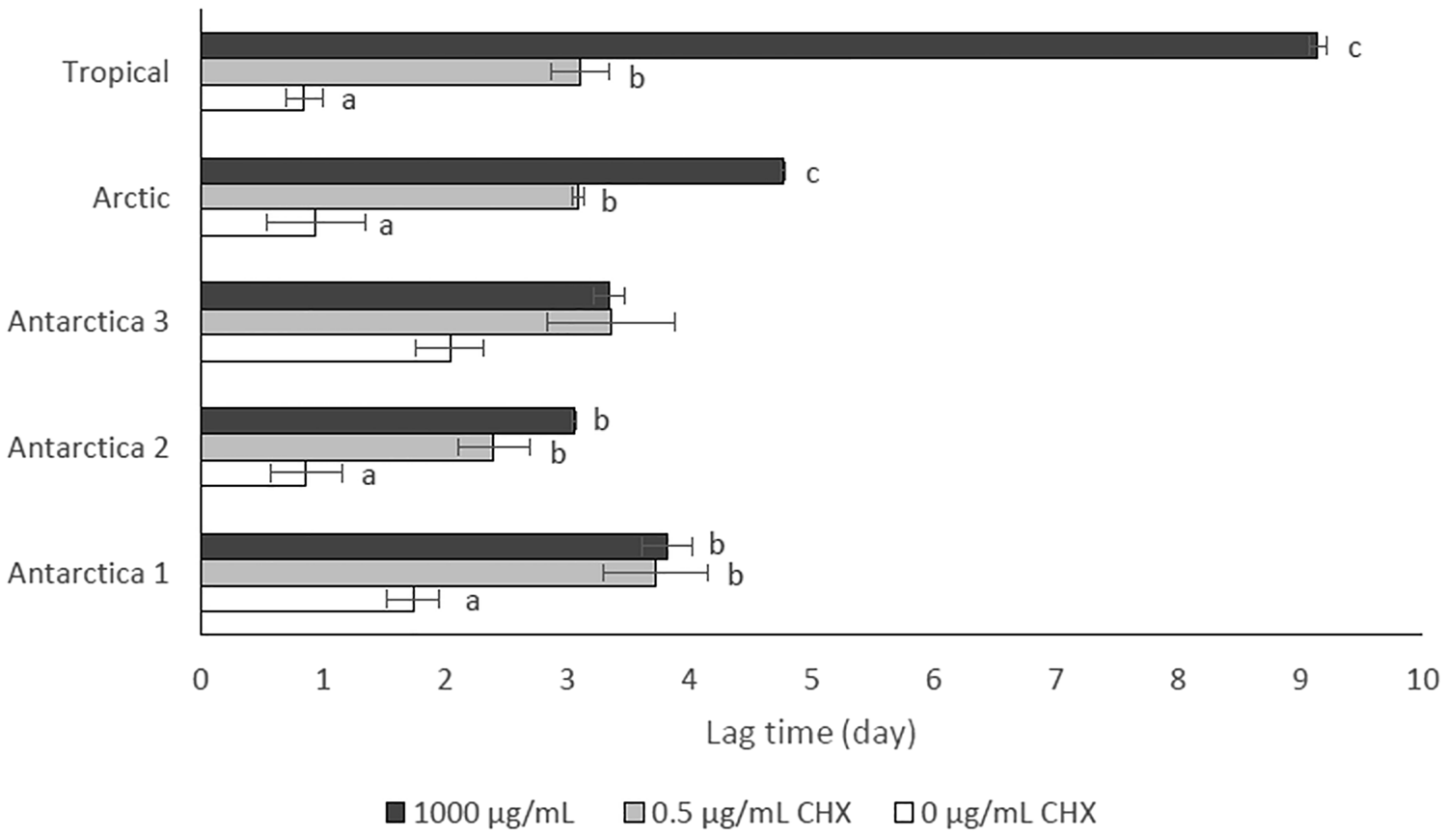
Mean (± standard error) of the lag time for each of five strains of *Stichococcus*-like algae to reach constant growth rate in control BBM, BBM + 0.5 µg/mL CHX and BBM + 1000 µg/mL CHX. Means with different letters (a, b, c) are significantly different (Tukey’s HSD, *p* < 0.05).

The times required to reach a constant growth rate by these strains were compared between BBM, BBM + 0.5 µg/mL CHX and BBM + 1000 µg/mL CHX (Fig. 3). Both low and high CHX concentrations had significant effects on the lag time of strain “Tropical”, but similar effects were not apparent in strains “Antarctica 1”, “Antarctica 2” and “Arctic”. Although post hoc testing identifies marginally non-significant differences in lag time between control and treatments in “Antarctica 3” (*p* = 0.06), possibly suggesting greater initial resistance to exposure to CHX, the overall shape of the response was visually very similar to that of strains “Antarctica 1” and “Antarctica 2”.

### Confirmation of generic assignment of *Stichococcus*-like algal strains

Morphological evaluations conducted on all six strains conform closely to the previous descriptions of *Stichococcus* Nageli 1849 (Fig. S2).

**Figure 4:**
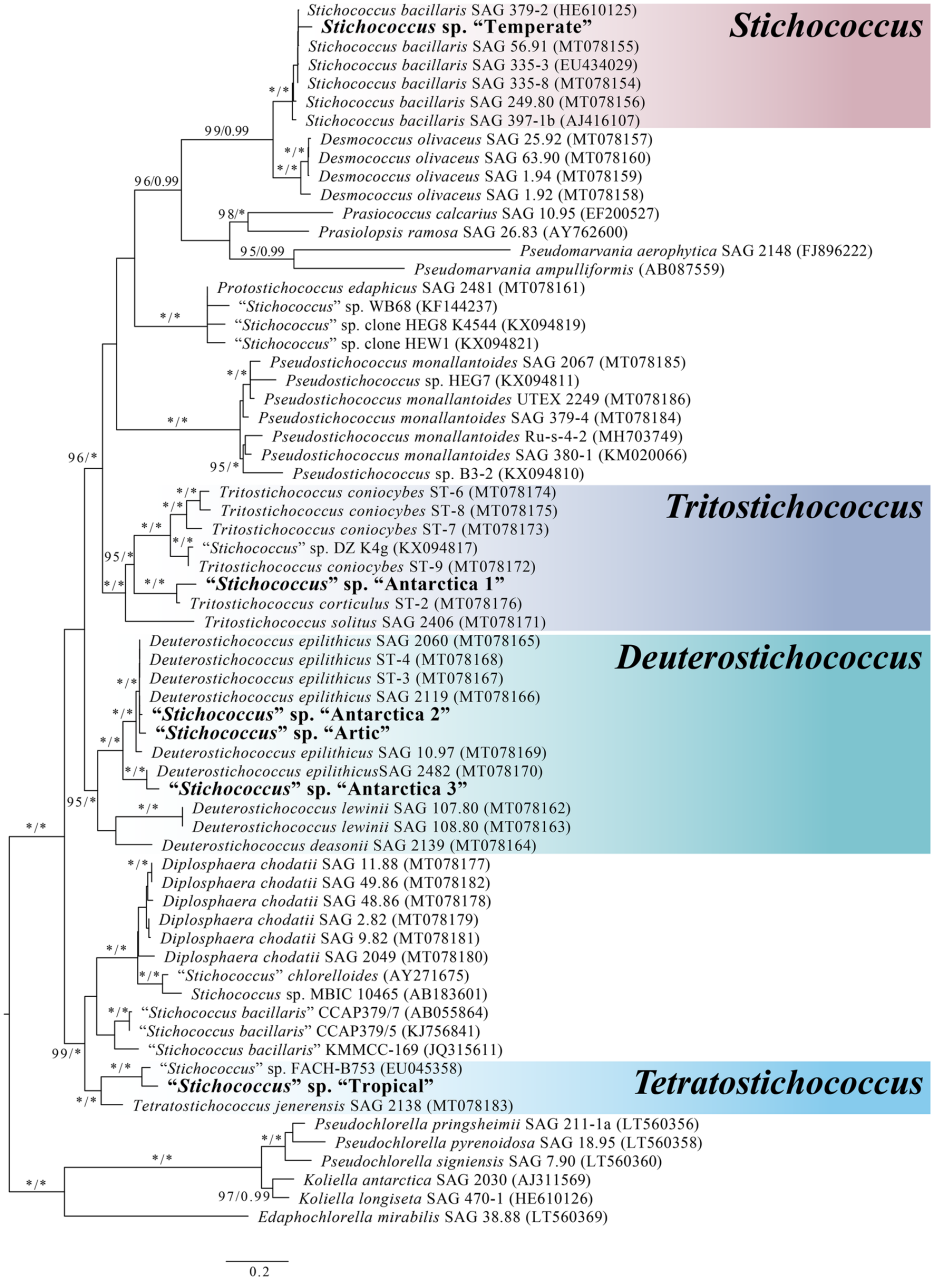
Maximum-likelihood tree based on concatenated 18S rDNA and ITS 2 sequences obtained in this study (in boldface). Numbers next to branches indicate statistical support value (maximum likelihood bootstrap/Bayesian posterior probabilities). *Chlorella*-like algal strains including *Pseudochlorella*, *Koliella* and *Edaphachlorella* were used as the outgroup. Scale bar represents 0.2 changes per site.

The sequence dataset consisted of concatenated 18S rDNA and ITS2 sequences (12,917 bp including gaps). Both ML and BI analyses yielded identical topologies hence only the ML tree is presented here (Fig. 4). “Temperate” was the only strain that can be confidently identified as *S. bacillaris*, located within a strongly supported clade (99/0.99 for ML and BI, respectively) containing confirmed strains of *S. bacillaris*^29^. The other *Stichococcus*-like strains were located within the newly erected clades^28^ with “Antarctica 1” being within the *Tritostichococcus* clade, “Antarctica 2”, “Antarctica 3” and “Arctic” clustered within the *Deuterostichococcus* clade and “Tropical” being within the *Tetratostichococcus* clade.

## Discussion

The data presented here conclusively demonstrate that multiple *Stichococcus*-like algal strains obtained across a global range of locations are able to grow in the presence of the widely used eukaryotic growth inhibitor cycloheximide. This is the first demonstration of chlorophytes being able to grow in the presence of this antibiotic. In susceptibility testing, it is common practice to use the performance standards provided by the Clinical Laboratory Standards Institute (CLSI)^30^ or European Committee on Antimicrobial Susceptibility Testing (EUCAST)^31^ as a guideline for MIC breakpoints in order to determine whether a strain is resistant, intermediate or susceptible to an antibiotic compound. However, the existing standards are limited to bacteria and fungi. From the standard MIC list for unicellular fungi (yeasts), most strains have MIC less than 10 µg/mL. This value is far below the MIC recorded in most microalgal strains in the present study (> 1000 µg/mL), and even considerably lower than the less resistant “Temperate” strain (62.5 µg/mL). Previously^8^, eukaryotic algae have been recorded as being completely inhibited at all concentrations of CHX.

In the present study strains “Antarctica 1”, “Antarctica 2”, “Antarctica 3”, “Arctic” and “Tropical” were highly resistant to CHX and exhibited an MIC > 1000 µg/mL. Most of these strains showed statistically indistinguishable growth rates, the only exception being between “Antarctica 1” treatment and “Tropical” control and treatment conditions, with even then this difference being small. The presence of CHX resulted in an extended lag period in each strain relative to their control, suggesting the cells were initially stressed by the treatment^32^. However, based on the growth rate of each strain, normal growth was then resumed. This temporary inhibition of cell division may be a form of physiological adaptation to the toxicants or due to death of susceptible cells^33^. The growth rates measured in the present study indicated that cells of all strains were able to grow normally once they had adapted to CHX. The lack of significant differences in the lag times of cultures exposed to the lowest and highest CHX concentrations in strains “Antarctica 1”, “Antarctica 2” and “Antarctica 3” suggested that growth was only affected by presence of CHX, not the concentration. Only the “Temperate” strain had a lower MIC of 62.5 µg/mL.

Resistance to high CHX concentration was also observed in the “Tropical” strain, isolated from tropical rainforest. Although this strain required nine days to adapt to 1000 µg/mL CHX (Fig. 3), the subsequent growth rate at this concentration was not significantly different from that of the control (Fig. 2). This response is analogous to previous studies in which growth of *Stichococcus* cells was recorded only after 4–5 days’ exposure to multiple herbicides^32^ or aluminum^33^.

Morphological combined with molecular genetic assessment of the six *Stichococcus*-like strains was able to place only one of the studied strains, “Temperate”, definitively within a known species. Its characteristics conformed to *S. bacillaris* Nägeli 1849. The species identity of the other five *Stichococcus*-like strains lie within independent lineages that have now been separated in a newly-available molecular phylogenetics analysis from the redefined *Stichococcus* clade^28^. The morphologically simple features that characterize *Stichococcus-*like strains restrict reliable identification of species within the genus.

Soils are a reservoir for many natural antibiotic compounds^34–37^. In Antarctica there is evidence that soil communities have yet to be exposed to the extensive microbial and chemical contamination that is now widespread on other continents^38,39^. There has not been extensive release of human manufactured antibiotics in the region and, hence, the presence of antibiotics is unlikely to be a human artefact. Polar soils are characteristically nutrient-limited^40^ and competition between microorganisms is likely to be an important stressor. It is therefore plausible that there has been evolution of antagonistic activity against potential competitors^41–43^. Accurate determination of antibiotic diversity and concentration in soil is difficult, especially when the compounds are made up of complex mixtures of small molecules with different properties^44^. Exposure to natural antibiotics is considered to be the major driver in the evolutionary selection for antibiotic resistance in the soil microbial community^45–47^. Although no such instances have been reported in microalgae, an analogous response is known in microalgae naturally exposed to heavy metals, which are another important environmental stressor^33,48^. The data presented here provide strong evidence of the potential for natural evolution of antibiotic resistance in eukaryotic algae.

Antibiotic resistance genes (ARGs) function to protect an organism from the inhibitory or harmful effects of an antibiotic produced by another organism. ARGs may evolve in nature in response to antibiotics produced by neighboring cells or be acquired through horizontal gene transfer^49^. Antibiotic resistance genes found in environments lacking human impact are potentially ancient genes that are transferred vertically from parent to offspring, with limited or no horizontal transfer between species^37^. This is supported by the present study in which all four strains from the polar regions and the tropical strain showed the highest resistance to CHX.

This investigation has confirmed and extended our preliminary observation of CHX resistance in eukaryotic Antarctic microalgae isolated from CHX-supplemented growth medium. Within the genera tested here, this novel antibiotic resistance was restricted to strains of *Stichococcus*-like algae from globally widespread locations*.*

## Methods

### Sample collection

Six *Stichococcus*-like algal strains were isolated from samples originating from Antarctica, the Arctic, temperate and tropical regions. Two control algae with susceptibility to low concentrations of CHX, *Chlorella* and *Coccomyxa*, were isolated from Antarctica. Details of each strain are presented in Table 2.

**Table 2:** Original collection localities of the six *Stichococcus*-like algal strains and the two control algae used in this study.

Strain	GenBank accession number		Location	Habitat	Year of isolation	Source
	18S	ITS-2				
“Antarctica 1”	MN968057	MN968730	Signy Island, Antarctica 60°43″S, 45°37″W	Soil	2016	Field material
“Antarctica 2”	MN968346	MN968731	Signy Island, Antarctica 60°43″S, 45°37″W	Soil	2016	Field material
“Antarctica 3”	MN968320	MN968556	Signy Island, Antarctica 60°43″S, 45°37″W	Soil	~ 1972	Culture Collection of Algae and Protozoa (CCAP)
“Arctic”	MN968500	MN968719	Prudhoe Bay, Alaska 70°19′32″N, 148°42′41″W	–	–	Culture Collection of Algae at University of Texas (UTEX)
“Temperate”	MN968499	MN968718	Bernburg, Germany 51°47′40″N, 11°44′24″E	Melt water	before 1936	Culture Collection of Algae at Göttingen University (SAG)
“Tropical”	MN968502	MN968732	Penang Hill, Malaysia 5°25′28″N, 100°16′08″E	Corticolous on tree *Fragea fragans*	2017	Field material
*Chlorella*	–	–	Signy Island, Antarctica 60°43″S, 45°37″W	Soil	2016	Field material
*Coccomyxa*	–	–	Signy Island, Antarctica 60°43″S, 45°37″W	Soil	2016	Field material

Cultures were established on 1% agarised full strength Bold’s Basal Medium (BBM)^50,51^. These were incubated under a cool white fluorescent lamp (12:12 h light:dark cycle at 27 µmol/m^2^/s) at 18 °C for polar strains and 24 °C for temperate and tropical strains.

### General experimental procedures

Seven days prior to the growth inhibition assay, cells of each strain were introduced to fresh BBM liquid medium to ensure that cultures were in exponential phase. CHX powder was added directly to BBM. The mixture was filter sterilized using an 0.22 µm microfilter (HmbG, cat no. P0376). Cultures of all strains were incubated at 24 °C under continuous light provided by white fluorescent lamps with 27 µmol/m^2^/s light intensity in a controlled temperature culture room.

### Microalgal growth inhibition assay

Serial dilutions of CHX were prepared by two-fold dilutions following the protocol of broth microdilution^52^, resulting in final concentrations of 1000, 500, 250, 125, 62.5, 31.3, 15.6, 8, 4, 2, 1 and 0.5 µg/mL. Three replicates were prepared at each concentration. The assay was carried out for 15 d using sterile, transparent, 96-well polystyrene microtitre plates (TPP, cat. no. 92096).

A week-old culture was adjusted to 1 × 10^3^ cells/mL and 100 µL was injected into three replicate wells at each CHX concentration and three replicate wells of a positive control of BBM without CHX. Plates were sealed using Parafilm® M in order to prevent evaporation and the plates were illuminated from above and below during incubation.

The growth of cells in the control and at each CHX concentration was assessed using three different methods: visual assessment, chlorophyll fluorescence and cell counts. Chlorophyll fluorescence was measured daily. Cultures were transferred from transparent to black 96-well microplates^53^. The cultures were mixed thoroughly by repeatedly loading in the pipette tips before transferring. Chlorophyll fluorescence was measured using a microplate reader (Tecan infinite M1000 PRO; Tecan Austria GmbH) with excitation wavelength of 485 nm and emission wavelength of 680 nm^53^. The fluorescence data were expressed as relative fluorescence units (RFU). Cell density was assessed only on the positive control using a Neubauer haemocytometer. Cells were counted from the four corner squares of the chamber and density calculated using the given formula^54^. Growth in the wells was visually assessed by the unaided eye on day 15.

The strength of the relationship between chlorophyll fluorescence and cell number was assessed using Pearson’s correlation. A regression analysis was conducted to determine cell density estimates derived from chlorophyll fluorescence of the treatments followed by one-way ANOVA. Differences were accepted as significant at *p* < 0.05. Post hoc analyses were performed using Tukey’s test for multiple comparisons of means. All statistical analyses were performed using SPSS.

Determinations were made of the minimum inhibitory concentration (MIC), population growth rate and lag time. The visual assessment at day 15 was used to determine MIC. Population growth rates were evaluated for strains that were resistant to up to 1000 µg/mL CHX. The daily relative fluorescence unit (RFU) measurements were used to calculate the population growth rate, α, of each strain in all three replicates of both the control and BBM + 1000 µg/mL CHX using the GrowthRates program^55^. This also estimated the duration of the lag phase by extrapolating the slope of the exponential phase back to the initial RFU. The generated growth rates and lag time of the control and treatments were compared using ANOVA. Differences were accepted as significant at *p* < 0.05.

### Morphological examination using light microscopy

Morphological assessment was made of all strains using an Olympus BX53 light microscope at 80–2000× magnification. Observations and measurements were made of cell shape, cell dimensions, size of chloroplast, presence of pyrenoid, formation and fragmentation of filaments and cell division. Size measurements were made on 30 randomly chosen cells. Photomicrographs were taken. Identification of morphospecies was carried out based on the relevant taxonomic literature^56–58^.

### Molecular analyses

DNA was extracted using the Tiangen DNAsecure Plant Kit (Beijing) following the manufacturer’s instructions. The extracted DNA was stored frozen at − 20 °C. The quality and purity of the extracted DNA were determined using a Nanodrop Quawell UV Spectrophotometer Q3000. The 18S rDNA gene and the internal transcribed spacer 2 (ITS-2) region were amplified using polymerase chain reaction (PCR) and the combination of primers 20F (5′-GTA GTC ATA TGC TTG TCT C-3′) and 18L (5′-CAC CTA CGG AAA CCT TGT TAC GAC TT-3′) for the 18S rRNA gene^59^ and primers ITS_f (5′-AGG AGA AGT CGT AAC AAG GT-3′) and ITS_r (5′-TCCTCCGCTTATTGATATGC-3′) for the ITS-2 region^60^. This resulted in products of approximately 1700 bp for the 18S rDNA and 300 bp for the ITS-2 region. The reaction mix comprised 2 μL of extracted DNA used in 50 μL reactions containing 1 μL of each forward and reverse primer, 21 μL of ultrapure water and 25 μL of *MyTaq*™ *Red Mix*, which is a pre-prepared mixture of buffer, dNTPs and Taq polymerase (Bioline, United Kingdom).

PCR was carried out using a Bio-Rad Thermal Cycler with standard parameters. Thermal cycling conditions to amplify the 18S region were set at 95 °C for 5 min for pre-denaturation, followed by 35 cycles of denaturation at 94 °C for 1 min, 56 °C for 1 min, 72 °C for 3 min with a final extension at 72 °C for 10 min. For the ITS-2 region, the conditions used were 96 °C for 5 min for pre-denaturation, followed by 40 cycles of denaturation at 96 °C for 1 min, 56 °C for 1 min, 72 °C for 1 min with a final extension at 72 °C for 5 min. Once the reaction was completed, the integrity of the PCR product was verified using a 2% agarose gel. Amplified DNA was purified using the MEGAquick-spinTM Total Fragment DNA Purification Kit (iNtRON Biotechnology, Korea).

### Phylogenetic tree analyses

All sequences were edited and assembled using the Geneious 11.0 software package (Biomatters, http://www.geneious.com). Sequence alignments were prepared using the MUSCLE algorithm in Geneious 11.0 and then manually checked by eye. The closest related sequences were identified from GenBank using the Basic Local Alignment Search Tool (BLAST) algorithm^61^.

The alignment, which included the sequences newly obtained in this study together with additional sequences of closely related species from GenBank, contained 73 sequences for the 18S rDNA analysis and 46 sequences for the ITS-2 analysis. All new sequences generated in this study have been deposited in GenBank under accession numbers listed in Table 2.

Phylogenetic analyses were conducted based on the concatenated 18S rDNA and ITS-2 dataset, using two different methods: maximum likelihood (ML) and Bayesian inference (BI). Before carrying out these analyses, the best-fit model of DNA substitution was determined using the program Kakusan4^62^. ML analyses were performed with RaxML v7^63^ in Geneious 11.0 using the general time-reversible invariant-sites (GTRI) nucleotide substitution model with the default parameters. The bootstrap probability of each branch was calculated using 1000 replications. BI analyses were performed using the program MrBayes v3.1.2^64^. Two independent analyses, each consisting of four Markov chains, were run simultaneously for 3,000,000 generations, sampling every 100 generations. A burn-in of 25% of saved trees was removed, and the remaining trees were used to calculate the Bayesian posterior probability values. ML and BI trees were edited with the program FigTree v1.3.1^65^. *Chlorella-*like strains were used as outgroup to root the tree.

## Supplementary Information

Supplementary Information.

## References

[1] D’Costa VM, King CE, Kalan L, Morar M, Sung WW, Schwarz C (2011). Antibiotic resistance is ancient. Nature.

[2] Kaur P, Peterson E (2018). Antibiotic resistance mechanisms in bacteria: Relationships between resistance determinants of antibiotic producers, environmental bacteria, and clinical pathogens. Front. Microbiol..

[3] Munita JM, Arias CA (2016). Mechanisms of antibiotic resistance. Microbiol. Spectr..

[4] Leach BE, Ford JH, Whiffen AJ (1947). Actidione, an antibiotic from *Streptomyces**griseus*. J. Am. Chem. Soc..

[5] Schneider-Poetsch T, Ju J, Eyler DE, Dang Y, Bhat S, Merrick WC (2010). Inhibition of eukaryotic translation elongation by cycloheximide and lactimidomycin. Nat. Chem. Biol..

[6] Whiffen AJ (1948). The production, assay, and antibiotic activity of actidione, an antibiotic from *Streptomyces**griseus*. J. Bacteriol..

[7] Palmer CM, Maloney TE (1955). Preliminary screening for potential algicides. Ohio J. Sci..

[8] Zehnder A, Hughes EO (1958). The antialgal activity of actidione. Can. J. Microbiol..

[9] Hunter EO, McVeigh I (1961). The effects of selected antibiotics on pure cultures of algae. Am. J. Bot..

[10] Vaara T, Vaara M, Niemelä S (1979). Two improved methods for obtaining axenic cultures of cyanobacteria. Appl. Environ. Microbiol..

[11] Castenholz RW (1988). Culturing methods for cyanobacteria. Methods Enzymol..

[12] Bolch CJ, Blackburn SI (1996). Isolation and purification of Australian isolates of the toxic cyanobacterium *Microcystis**aeruginosa* Kützing. J. Appl. Phychol..

[13] Choi GG, Bae MS, Ahn CY, Oh HM (2008). Induction of axenic culture of *Arthrospira**(Spirulina)**platensis* based on antibiotic sensitivity of contaminating bacteria. Biotechnol. Lett..

[14] Katoh H, Furukawa J, Tomita-Yokotani K, Nishi Y (2008). Isolation and purification of an axenic diazotrophic drought-tolerant cyanobacterium, *Nostoc**commune*, from natural cyanobacterial crusts and its utilization for field research on soils polluted with radioisotopes. Biochim. Biophys. Acta..

[15] Mutoh E, Ohta A, Takagi M (1998). Studies on cycloheximide-sensitive and cycloheximide-resistant ribosomes in the yeast *Candida**maltosa*. Gene.

[16] Takagi M, Kawai S, Shibuya I, Miyazaki M, Yano K (1986). Cloning in *Saccharomyces**cerevisiae* of a cycloheximide resistance gene from the *Candida**maltosa* genome which modifies ribosomes. J. Bacteriol..

[17] Dehoux P, Davies J, Cannon M (1993). Natural cycloheximide resistance in yeast: The role of ribosomal protein L41. Eur. J. Biochem..

[18] Adoutte-Panvier A, Davies JE (1984). Studies of ribosomes of yeast species: Susceptibility to inhibitors of protein synthesis *in**vivo* and *in**vitro*. Mol. Gen. Genet..

[19] Yagisawa F, Nishida K, Okano Y, Minoda A, Tanaka K, Kuroiwa T (2004). Isolation of cycloheximide-resistant mutants of *Cyanidioschyzon**merolae*. Cytologia.

[20] Thomas DN, Fogg GE, Convey P, Fritsen CH, Gili JM, Gradinger R (2008). The Biology of Polar Regions.

[21] Hughes KA (2006). Solar UV-B radiation, associated with ozone depletion, inhibits the Antarctic terrestrial microalga, *Stichococcus**bacillaris*. Polar Biol..

[22] Karsten U, Holzinger A (2014). Green algae in alpine biological soil crust communities: Acclimation strategies against ultraviolet radiation and dehydration. Biodivers. Conserv..

[23] Shekh RM, Singh P, Singh SM, Roy U (2011). Antifungal activity of Arctic and Antarctic bacteria isolates. Polar Biol..

[24] Wiser MJ, Lenski RE (2015). A comparison of methods to measure fitness in *Escherichia**coli*. PLoS ONE.

[25] Fritsch FE (1959). The Structure and Reproduction of the Algae.

[26] Fukushima H (1963). Studies on cryophytes in Japan. J. Yokohama Munic Univ. Ser. C.

[27] Hoham RW (1973). Pleiomorphism in the snow alga, *Raphidonema**nivale* Lagerh (Chlorophyta), and a revision of the genus *Raphidonema* Lagerh. Syesis.

[28] Pröschold T, Darienko T (2020). The green puzzle Stichococcus (Trebouxiophyceae, Chlorophyta): New generic and species concept among this widely distributed genus. Phytotaxa.

[29] Hodač L, Hallmann C, Spitzer K, Elster J, Faßhauer F, Brinkmann N (2016). Widespread green algae *Chlorella* and *Stichococcus* exhibit polar-temperate and tropical-temperate biogeography. FEMS Microbiol. Ecol..

[30] Clinical and Laboratory Standards Institute (2017). Performance Standards for Antimicrobial Susceptibility Testing: 27th Informational Supplement.

[31] European Committee for Antimicrobial Susceptibility Testing (EUCAST) of the European Society of Clinical Microbiology and Infectious Diseases (ESCMID) (2003). EUCAST discussion document E.Dis. 5.1. Determination of minimum inhibitory concentrations (MIC’s) of antibacterial agents by broth dilution. Clin. Microbiol. Infect..

[32] Bertrand RL (2019). Lag phase is a dynamic, organized, adaptive, and evolvable period that prepares bacteria for cell division. J. Bacteriol..

[33] Claesson A, Törnqvist L (1988). The toxicity of aluminium to two acido-tolerant green algae. Water Res..

[34] Knapp CW, McCluskey SM, Singh BK, Campbell CD, Hudson G, Graham DW (2011). Antibiotic resistance gene abundances correlate with metal and geochemical conditions in archived Scottish soils. PLoS ONE.

[35] Su JQ, Wei B, Xu CY, Qiao M, Zhu YG (2014). Functional metagenomic characterization of antibiotic resistance genes in agricultural soils from China. Environ. Int..

[36] Tomova I, Stoilova-Disheva M, Lazarkevich I, Vasileva-Tonkova E (2015). Antibiotic activity and resistance to heavy metals and antibiotics of heterotrophic bacteria isolated from sediment and soil samples collected from two Antarctic islands. Front. Life Sci..

[37] Van Goethem MW, Pierneef R, Bezuidt OK, Van De Peer Y, Cowan DA, Makhalanyane TP (2018). A reservoir of ‘historical’ antibiotic resistance genes in remote pristine Antarctic soils. Microbiome.

[38] Cowan DA, Chown SL, Convey P, Tuffin M, Hughes K, Pointing S, Vincent WF (2011). Non-indigenous microorganisms in the Antarctic: Assessing the risks. Trends Microbiol..

[39] Convey P, Chown SL, Clarke A, Barnes DK, Bokhorst S, Cummings V (2014). The spatial structure of Antarctic biodiversity. Ecol. Monogr..

[40] Cowan DA, Makhalanyane TP, Dennis PG, Hopkins DW (2014). Microbial ecology and biogeochemistry of continental Antarctic soils. Front. Microbiol..

[41] Lo Giudice A, Bruni V, Michaud L (2007). Characterization of Antarctic psychrotrophic bacteria with antibacterial activities against terrestrial microorganisms. J. Basic Microbiol..

[42] Bell T, Callender K, Whyte L, Greer C (2013). Microbial competition in polar soils: A review of an understudied but potentially important control on productivity. Biology.

[43] Núñez-Montero K, Barrientos L (2018). Advances in Antarctic research for antibiotic discovery: A comprehensive narrative review of bacteria from Antarctic environments as potential sources of novel antibiotic compounds against human pathogens and microorganisms of industrial importance. Antibiotics.

[44] Davies J (2006). Are antibiotics naturally antibiotics?. J. Ind. Microbiol. Biotechnol..

[45] Levy SB (2001). Antibiotic resistance: Consequences of inaction. Clin. Infect. Dis..

[46] Marshall BM, Levy SB (2011). Food animals and antimicrobials: Impacts on human health. Clin. Microbiol. Rev..

[47] Andersson DI, Hughes D (2012). Evolution of antibiotic resistance at non-lethal drug concentrations. Drug Resist. Updates.

[48] Kuwabara JS, Leland HV (1986). Adaptation of *Selenastrum**capricornutum* (Chlorophyceae) to copper. Environ. Toxicol. Chem..

[49] Martínez JL, Coque TM, Baquero F (2015). What is a resistance gene? Ranking risk in resistomes. Nat. Rev. Microbiol..

[50] Bold HC (1949). The morphology of *Chlamydomonas**chlamydogama* sp. nov. Bull. Torrey Bot. Club.

[51] Bischoff H, Bold HC (1963). Phycological studies IV. Some soil algae from enchanted rock and related algal species. Univ. Texas Publ..

[52] Balouiri M, Sadiki M, Ibnsouda SK (2016). Methods for in vitro evaluating antibiotic activity: A review. J. Pharm. Anal..

[53] Zhao Q, Chen AN, Hu SX, Liu Q, Chen M, Liu L, Shao CL, Tang XX, Wang CY (2018). Microalgal microscale model for microalgal growth inhibition evaluation of marine natural products. Sci. Rep..

[54] LeGresley M, McDermott G, Karlson B, Cusack C, Bresnan E (2010). Counting chamber methods for quantitative phytoplankton analysis—Haemocytometer, Palmer-Maloney cell and Sedgewick-Rafter cell. Microscopic and Molecular Methods for Quantitative Phytoplankton Analysis.

[55] Hall BG, Acar H, Nandipati A, Barlow M (2010). Growth rates made easy. Mol. Biol. Evol..

[56] Mattox KR, Bold HC (1962). Phycological studies III. The taxonomy of certain ulotrichacean algae. Univ. Texas Publ..

[57] Prescott GW (2010). Algae of the Western Great Lakes area.

[58] John DM, Whitton BA, Brook AJ (2011). The Freshwater Algal Flora of the British Isles.

[59] Hallmann C, Stannek L, Fritzlar D, Hause-Reitner D, Friedl T, Hoppert M (2013). Molecular diversity of phototrophic biofilms on building stone. FEMS Microbiol. Ecol..

[60] Liu J, Gerken H, Li Y (2014). Single-tube colony PCR for DNA amplification and transformant screening of oleaginous microalgae. J. Appl. Phycol..

[61] Altschul SF, Madden TL, Schäffer AA, Zhang J, Zhang Z, Miller W (1997). Gapped BLAST and PSI-BLAST: A new generation of protein database search programs. Nucleic Acids Res..

[62] Kanehisa M, Goto S, Sato Y, Furumichi M, Tanabe M (2011). KEGG for integration and interpretation of large-scale molecular data sets. Nucleic Acids Res..

[63] Stamatakis A (2006). RAxML-VI-HPC: Maximum likelihood-based phylogenetic analyses with thousands of taxa and mixed models. Bioinformatics.

[64] Ronquist F, Huelsenbeck JP (2003). MrBayes 3: Bayesian phylogenetic inference under mixed models. Bioinformatics.

[65] Rambaut, A. *FigTree*. Version 1.3.1 [software]. Available from: http://www.treebioedacuk/software/figtree (2009).

