# Supplementary material for: Strong and widespread cycloheximide resistance in Stichococcus-like eukaryotic algal taxa
Source: Sci Rep. 2022 Jan 20;12:1080. doi: 10.1038/s41598-022-05116-y (PMC8776791; doi:10.1038/s41598-022-05116-y)
Supplement: Supplementary file 1 — Supplementary Information. [file 41598_2022_5116_MOESM1_ESM.pdf]

# Supplementary Information

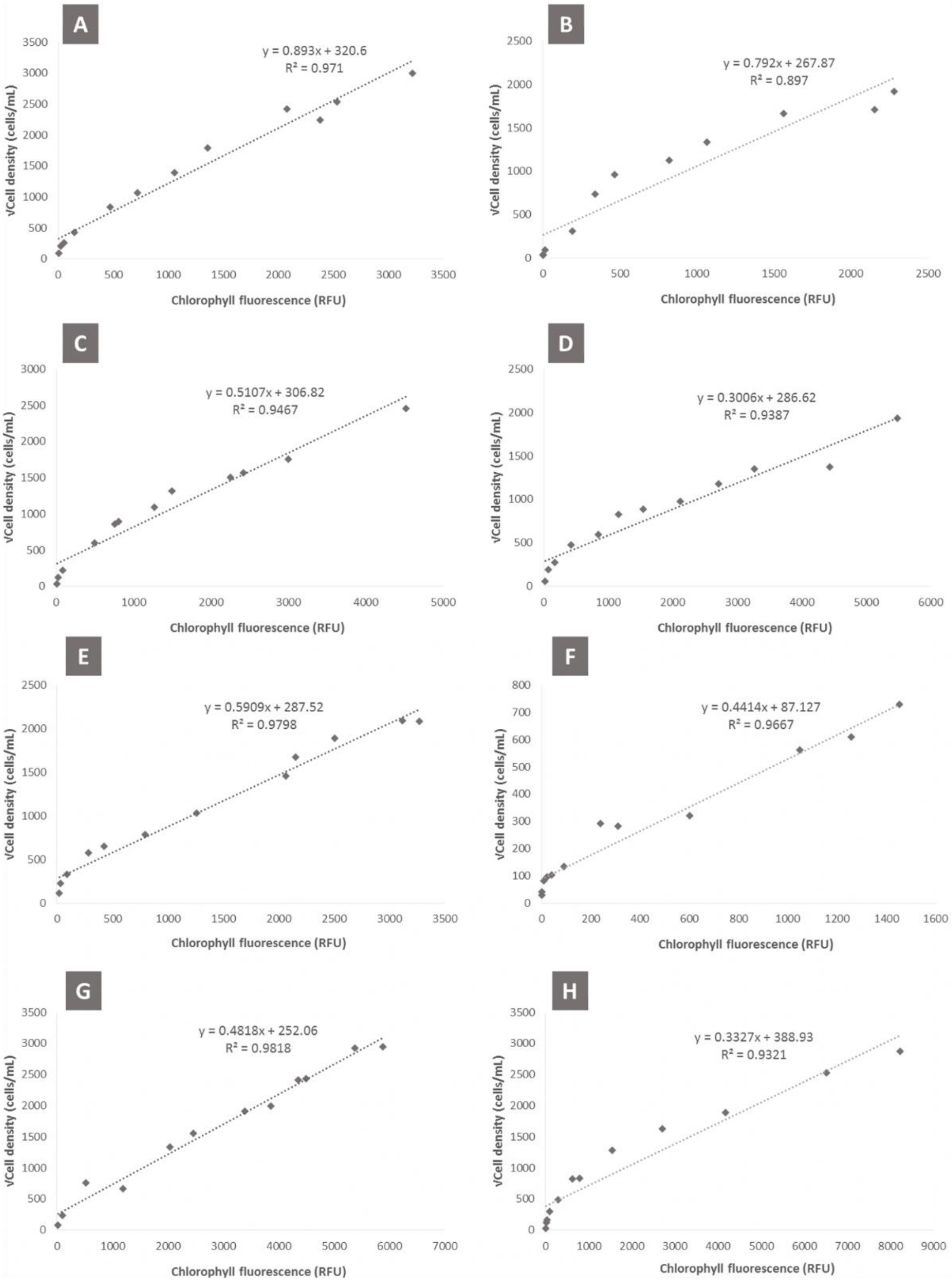


**S1 Fig. Cell density as a function of chlorophyll fluorescence of control cultures**. A – F, six strains of *Stichococcus*; A, "Antarctica 3"; B, "Antarctica 1"; C, "Antarctica 2"; D, "Arctic"; E, "Tropical"; F, "Temperate"; G, *Chlorella*; H, *Coccomyxa*.

**S1 Table. Pearson's correlation between the measurements of chlorophyll fluorescence and cell density for six strains of *Stichococcus* and two control strains.** Values with asterisk (**) indicate significant correlation at $p < 0.01$ (2-tailed) for all strains.

| **Strain** | **Pearson's correlation coefficient (r)** |
|---|---|
| **"Antarctica 1"** | 0.947* |
| **"Antarctica 2"** | 0.973* |
| **"Antarctica 3"** | 0.985* |
| **"Arctic"** | 0.969* |
| **"Tropical"** | 0.990* |
| **"Temperate"** | 0.983* |
| ***Chlorella*** | 0.991* |
| ***Coccomyxa*** | 0.965* |

**S2 Table. Welch's ANOVA of combined variables and multiple comparison of post-hoc test (Games Howell).**

| **Robust Tests of Equality of Means** | | | | |
|---|---|---|---|---|
| Growth rate | | | | |
| | Statistic* | df1 | df2 | Sig. |
| Welch | 8.784 | 9 | 7.747 | .003 |
| a. Asymptotically F distributed. | | | | |

| Multiple Comparisons | | | | | | |
|---|---|---|---|---|---|---|
| Dependent Variable: Growth rate | | | | | | |
| **Games-Howell** | | | | | | |
| Combination Variable | Combination Variable | Mean Difference (I-J) | Std. Error | Sig. | 95% Confidence Interval | |
| | | | | | Lower Bound | Upper Bound |
| “Antarctica 1” {control) | “Antarctica 1”-1000 | -.04999 | .03522 | .867 | -.2752 | .1752 |
| | “Antarctica 2”-0 | .06474 | .04311 | .844 | -.1738 | .3033 |
| | “Antarctica 2”-1000 | .03499 | .03195 | .948 | -.2273 | .2973 |
| | “Antarctica 3”-0 | -.03678 | .04263 | .989 | -.2728 | .1992 |
| | “Antarctica 3”-1000 | .02245 | .03073 | .993 | -.2730 | .3179 |
| | “Arctic”-0 | .05360 | .04898 | .959 | -.2251 | .3324 |
| | “Arctic”-1000 | -.04390 | .07859 | .999 | -.6185 | .5306 |
| | “Tropical”-0 | .10100 | .03233 | .349 | -.1539 | .3559 |
| | “Tropical”-1000 | .10956 | .03238 | .302 | -.1446 | .3637 |
| “Antarctica 1” (treatment) | “Antarctica 1”-0 | .04999 | .03522 | .867 | -.1752 | .2752 |
| | “Antarctica 2”-0 | .11473 | .03511 | .279 | -.1094 | .3389 |
| | “Antarctica 2”-1000 | .08498 | .01988 | .156 | -.0461 | .2161 |
| | “Antarctica 3”-0 | .01321 | .03451 | 1.000 | -.2050 | .2314 |
| | “Antarctica 3”-1000 | .07244 | .01785 | .241 | -.0902 | .2351 |
| | “Arctic”-0 | .10360 | .04211 | .493 | -.1938 | .4010 |
| | “Arctic”-1000 | .00609 | .07450 | 1.000 | -.6446 | .6568 |
| | “Tropical”-0 | .15099* | .02049 | .031 | .0229 | .2791 |
| | “Tropical”-1000 | .15956* | .02055 | .026 | .0316 | .2875 |
| “Antarctica 2” (control) | “Antarctica 1”-0 | -.06474 | .04311 | .844 | -.3033 | .1738 |
| | “Antarctica 1”-1000 | -.11473 | .03511 | .279 | -.3389 | .1094 |
| | “Antarctica 2”-1000 | -.02975 | .03183 | .975 | -.2908 | .2313 |
| | “Antarctica 3”-0 | -.10152 | .04254 | .484 | -.3370 | .1339 |
| | “Antarctica 3"-1000 | -.04229 | .03061 | .868 | -.3365 | .2519 |
| | "Arctic"-0 | -.01114 | .04891 | 1.000 | -.2897 | .2674 |
| | "Arctic"-1000 | -.10864 | .07854 | .874 | -.6838 | .4665 |
| | "Tropical"-0 | .03626 | .03221 | .942 | -.2173 | .2898 |
| | "Tropical"-1000 | .04483 | .03226 | .870 | -.2080 | .2976 |
| "Antarctica 2" | "Antarctica 1"-0 | -.03499 | .03195 | .948 | -.2973 | .2273 |

| | | | | | | |
|---|---|---|---|---|---|---|
| (Treatment) | "Antarctica 1"-1000 | -.08498 | .01988 | .156 | -.2161 | .0461 |
| | "Antarctica 2"-0 | .02975 | .03183 | .975 | -.2313 | .2908 |
| | "Antarctica 3"-0 | -.07177 | .03117 | .557 | -.3255 | .1820 |
| | "Antarctica 3"-1000 | -.01254 | .00996 | .909 | -.0900 | .0649 |
| | "Arctic"-0 | .01861 | .03942 | 1.000 | -.3249 | .3621 |
| | "Arctic"-1000 | -.07890 | .07301 | .947 | -.7737 | .6159 |
| | "Tropical"-0 | .06601 | .01415 | .090 | -.0130 | .1450 |
| | "Tropical"-1000 | .07457 | .01425 | .062 | -.0051 | .1542 |
| "Antarctica 3" (control) | "Antarctica 1"-0 | .03678 | .04263 | .989 | -.1992 | .2728 |
| | "Antarctica 1"-1000 | -.01321 | .03451 | 1.000 | -.2314 | .2050 |
| | "Antarctica 2"-0 | .10152 | .04254 | .484 | -.1339 | .3370 |
| | "Antarctica 2"-1000 | .07177 | .03117 | .557 | -.1820 | .3255 |
| | "Antarctica 3"-1000 | .05923 | .02992 | .670 | -.2280 | .3465 |
| | "Arctic"-0 | .09038 | .04848 | .695 | -.1874 | .3681 |
| | "Arctic"-1000 | -.00712 | .07827 | 1.000 | -.5858 | .5716 |
| | "Tropical"-0 | .13778 | .03156 | .183 | -.1086 | .3842 |
| | "Tropical"-1000 | .14635 | .03161 | .161 | -.0993 | .3920 |
| "Antarctica 3" (Treatment) | "Antarctica 1"-0 | -.02245 | .03073 | .993 | -.3179 | .2730 |
| | "Antarctica 1"-1000 | -.07244 | .01785 | .241 | -.2351 | .0902 |
| | "Antarctica 2"-0 | .04229 | .03061 | .868 | -.2519 | .3365 |
| | "Antarctica 2"-1000 | .01254 | .00996 | .909 | -.0649 | .0900 |
| | "Antarctica 3"-0 | -.05923 | .02992 | .670 | -.3465 | .2280 |
| | "Arctic"-0 | .03115 | .03844 | .987 | -.3422 | .4045 |
| | "Arctic"-1000 | -.06635 | .07248 | .975 | -.7796 | .6469 |
| | "Tropical"-0 | .07855 | .01112 | .068 | -.0117 | .1688 |
| | "Tropical"-1000 | .08711 | .01125 | .056 | -.0045 | .1788 |
| "Arctic" (control) | "Antarctica 1"-0 | -.05360 | .04898 | .959 | -.3324 | .2251 |
| | "Antarctica 1"-1000 | -.10360 | .04211 | .493 | -.4010 | .1938 |
| | "Antarctica 2"-0 | .01114 | .04891 | 1.000 | -.2674 | .2897 |
| | "Antarctica 2"-1000 | -.01861 | .03942 | 1.000 | -.3621 | .3249 |
| | "Antarctica 3"-0 | -.09038 | .04848 | .695 | -.3681 | .1874 |
| | "Antarctica 3"-1000 | -.03115 | .03844 | .987 | -.4045 | .3422 |
| | "Arctic"-1000 | -.09751 | .08191 | .933 | -.6403 | .4453 |
| | "Tropical"-0 | .04740 | .03973 | .924 | -.2885 | .3833 |
| | "Tropical"-1000 | .05596 | .03977 | .863 | -.2792 | .3911 |
| "Arctic" (Treatment) | "Antarctica 1"-0 | .04390 | .07859 | .999 | -.5306 | .6185 |
| | "Antarctica 1"-1000 | -.00609 | .07450 | 1.000 | -.6568 | .6446 |
| | "Antarctica 2"-0 | .10864 | .07854 | .874 | -.4665 | .6838 |
| | "Antarctica 2"-1000 | .07890 | .07301 | .947 | -.6159 | .7737 |
| | "Antarctica 3"-0 | .00712 | .07827 | 1.000 | -.5716 | .5858 |
| | "Antarctica 3"-1000 | .06635 | .07248 | .975 | -.6469 | .7796 |
| | "Arctic"-0 | .09751 | .08191 | .933 | -.4453 | .6403 |

| | | | | | | |
|---|---|---|---|---|---|---|
| | "Tropical"-0 | .14490 | .07318 | .669 | -.5443 | .8341 |
| | "Tropical"-1000 | .15347 | .07320 | .631 | -.5351 | .8420 |
| "Tropical" (control) | "Antarctica 1"-0 | -.10100 | .03233 | .349 | -.3559 | .1539 |
| | "Antarctica 1"-1000 | -.15099* | .02049 | .031 | -.2791 | -.0229 |
| | "Antarctica 2"-0 | -.03626 | .03221 | .942 | -.2898 | .2173 |
| | "Antarctica 2"-1000 | -.06601 | .01415 | .090 | -.1450 | .0130 |
| | "Antarctica 3"-0 | -.13778 | .03156 | .183 | -.3842 | .1086 |
| | "Antarctica 3"-1000 | -.07855 | .01112 | .068 | -.1688 | .0117 |
| | "Arctic"-0 | -.04740 | .03973 | .924 | -.3833 | .2885 |
| | "Arctic"-1000 | -.14490 | .07318 | .669 | -.8341 | .5443 |
| | "Tropical"-1000 | .00857 | .01508 | .999 | -.0749 | .0920 |
| "Tropical" (Treatment)v | "Antarctica 1"-0 | -.10956 | .03238 | .302 | -.3637 | .1446 |
| | "Antarctica 1"-1000 | -.15956* | .02055 | .026 | -.2875 | -.0316 |
| | "Antarctica 2"-0 | -.04483 | .03226 | .870 | -.2976 | .2080 |
| | "Antarctica 2"-1000 | -.07457 | .01425 | .062 | -.1542 | .0051 |
| | "Antarctica 3"-0 | -.14635 | .03161 | .161 | -.3920 | .0993 |
| | "Antarctica 3"-1000 | -.08711 | .01125 | .056 | -.1788 | .0045 |
| | "Arctic"-0 | -.05596 | .03977 | .863 | -.3911 | .2792 |
| | "Arctic"-1000 | -.15347 | .07320 | .631 | -.8420 | .5351 |
| | "Tropical"-0 | -.00857 | .01508 | .999 | -.0920 | .0749 |

*. The mean difference is significant at the 0.05 level.

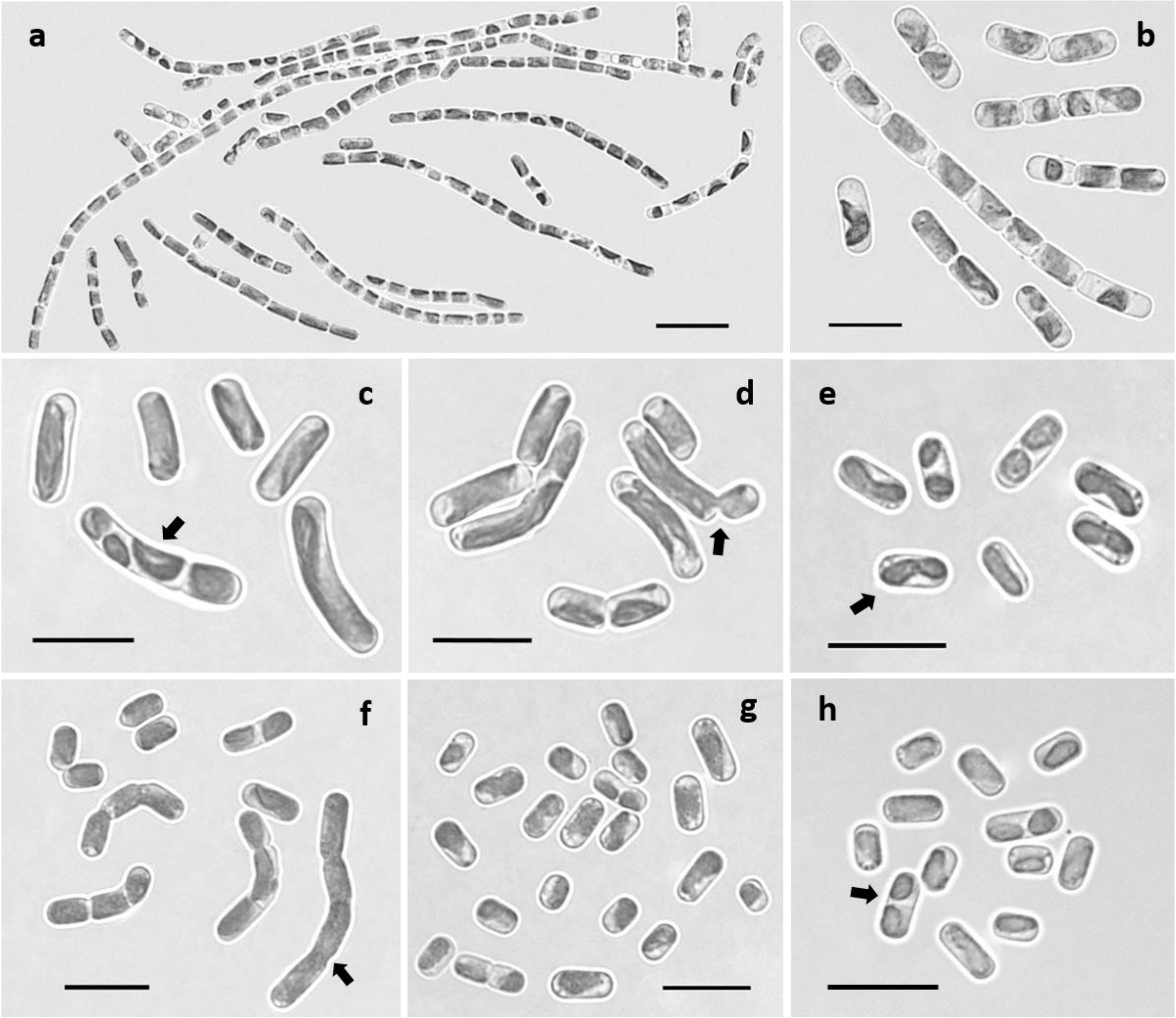


**S2 Fig. *Stichococcus* strains from different geographic regions grown in BBM medium.** "Temperate": a, b, cells forming filaments; "Arctic" : c, long cell with multiple chloroplasts (arrow), d, unequal cell division (arrow); "Antarctica 3": e, cells with cleaving chloroplast (arrow); "Antarctica 2": f, cell divided into multiple equal parts (arrow); "Antarctica 1": g, cells solitary and in pairs; "Tropical": h, chloroplast separated to cell poles as cell is preparing to divide (arrow). Scale bars: 10 µm for b – h; 20 µm for a.
